# Significance of Oxygen Supply in Jarosite Biosynthesis Promoted by *Acidithiobacillus ferrooxidans*

**DOI:** 10.1371/journal.pone.0120966

**Published:** 2015-03-25

**Authors:** Qingjie Hou, Di Fang, Jianru Liang, Lixiang Zhou

**Affiliations:** College of Resources and Environmental Sciences, Nanjing Agricultural University, Nanjing, 210095, China; Loyola University Chicago, UNITED STATES

## Abstract

Jarosite [(Na^+^, K^+^, NH_4_^+^, H_3_O^+^)Fe_3_(SO_4_)_2_(OH)_6_] is an efficient scavenger for trace metals in Fe- and SO_4_^2-^-rich acidic water. During the biosynthesis of jarosite promoted by *Acidithiobacillus ferrooxidans*, the continuous supply of high oxygen levels is a common practice that results in high costs. To evaluate the function of oxygen in jarosite production by *A*. *ferrooxidans*, three groups of batch experiments with different oxygen supply levels (i.e., loading volume percentages of FeSO_4_ solution of 20%, 40%, and 70% v/v in the flasks), as well as three groups of sealed flask experiments with different limiting oxygen supply conditions (i.e., the solutions were not sealed at the initial stage of the ferrous oxidation reaction by paraffin but were rather sealed at the end of the ferrous oxidation reaction at 48 h), were tested. The formed Fe-precipitates were characterized via X-ray powder diffraction and scanning electron microscope-energy dispersive spectral analysis. The results showed that the biosynthesis of jarosite by *A*. *ferrooxidans* LX5 could be achieved at a wide range of solution loading volume percentages. The rate and efficiency of the jarosite biosynthesis were poorly correlated with the concentration of dissolved oxygen in the reaction solution. Similar jarosite precipitates, expressed as KFe_3_ (SO_4_) _2_(OH)_6_ with Fe/S molar ratios between 1.61 and 1.68, were uniformly formed in unsealed and 48 h sealed flasks. These experimental results suggested that the supply of O_2_ was only essential in the period of the oxidation of ferrous iron to ferric but was not required in the period of ferric precipitation.

## Introduction

Jarosite [(Na^+^, K^+^, NH_4_^+^, H_3_O^+^)Fe_3_(SO_4_)_2_(OH)_6_] is a ubiquitous Fe(III)-mineral in acid mine drainage (AMD), AMD impacted sediments, and coastal acid sulfate soils [1,2]. In these natural acidic environments, the formation of jarosite is mainly attributed to the biological oxidation of Fe(II) to Fe(III) (by acidophilic Fe-oxidizing microorganisms) and precipitation of Fe(III) [3,4].

$$\begin{array}{l} 4Fe^{2+}+O_{2}+4H^{+}\to 4Fe^{3+}+2H_{2}O\\ 3Fe^{3+}+M^{+}+2SO_{4}{}^{2-}+6H_{2}O\to MFe_{3}{\left(SO_{4}\right)}_{2}{\left(OH\right)}_{6}+6H^{+}\\ \left(M=K^{+},NH_{4}{}^{+},Na^{+},H_{3}O^{+}\right) \end{array}$$

An important environmental function of this ferric hydroxysulfate mineral is its high capability to remove trace elements from solution via sorption or co-precipitation mechanisms [5]. The sorption capacity of K-jarosite [KFe_3_(SO_4_)_2_(OH)_6_] for Cr (VI) has been shown to reach up to 80 mg/g [6]. Jarosite, which functions as an iron scavenger, is widely used for the removal of iron impurities in hydrometallurgy [7,8]. Many investigations focusing on the field of AMD have indicated that a number of metal contaminants (Cu, As, Cd, Ni, etc.) in AMD-polluted sites can be naturally attenuated by newly formed jarosite precipitates [9,10]. Moreover, this material is also an efficient mineralogical control on aqueous concentrations of As in As-contaminated water, such as AMD and As-bearing groundwater [11]. Meanwhile, the formation of jarosite should be minimized as much as possible in several applications involving Fe-oxidizing microorganisms, such as ore biohydrometallurgy and sludge bioleaching, because jarosite decreases the solubilization efficiencies of targeted metals to an extent [12,13]. Given the high metal uptake feature of jarosite, the manner by which to synthesize this Fe(III)-mineral efficiently and economically has received a great deal of attention.

*Acidithiobacillus ferrooxidans*, one of the most common acidophilic Fe-oxidizing microorganisms, has been proven to be capable of forming various jarosite group minerals [2,3]. The formation rate and efficiency of jarosite by *A*. *ferrooxidans* are dependent on many factors, including pH, temperature, aging time, dissolved oxygen (DO) levels, concentrations of Fe^2+^ and monovalent cations (K^+^, NH_4_^+^, Na^+^ and H_3_O^+^), and molar ratio of Fe/K, Fe/NH_4_, Fe/Na, and Fe/H_3_O [14–16]. Previous studies have suggested that yellowish, well-crystalline NH_4_-jarosite could be synthesized within 48 h from acidic FeSO_4_ solutions inoculated with *A*. *ferrooxidans* at ambient temperature and pressure conditions [3,17]. NH4 in the crystal lattices of jarosite can be displaced by Ag^+^, K^+^, Na^+^, and H_3_O^+^ to form the corresponding jarosite group minerals [18,19]. The pH range of 1.0–3.0 is well-documented to favor jarosite formation, whereas a higher pH range results in the dominant formation of schwertmannite [Fe_8_O_8_(OH)_6_SO_4_] [11].

Given that *A*. *ferrooxidans* is known to be an aerobic bacterium, the continuous supply of O_2_ (e.g., DO > 5.0 mg/L) has been considered to be essential for jarosite synthesis, which will result in high costs [20,21]. Notably, our recent study found that when statically incubating FeSO_4_ solution containing *A*. *ferrooxidans* LX5 for 40 h, a significant amount of yellowish hydroxysulfate precipitates was generated, thereby implying that oxygen might not be indispensable throughout the experiment on jarosite biosynthesis [13]. However, this assumption is not supported by direct evidence. In particular, the quantitative relationship between DO level and formed jarosite amount remains unclear.

The objective of this study was to investigate the influence of DO on jarosite formation by *A*. *ferrooxidans*. The varying DO levels were indirectly achieved by changing the reaction solution loading volumes in the conical flasks as well as sealing the solution with paraffin at different reaction periods. The expected results obtained from this study will contribute to a better understanding of the biogenic jarosite formation process promoted by *A*. *ferrooxidans* in acidic natural or artificial environments while providing an economical approach for the highly efficient biosynthesis of jarosite.

## Materials and Methods

### Ethics statement

No specific permits were required for the described field studies, and no specific permissions were required for these locations. The location is not privately owned or protected in any way.

### Preparation of *A*. *ferrooxidans* LX5 cell suspension

*A*. *ferrooxidans* LX5 (CGMCC No. 0727) obtained from China General Microbiological Culture Collection Center (CGMCC) was grown in 9K medium developed by Silverman and Lundgren [22]. The medium contained the following mineral salts: 44.48 g of FeSO_4_·7H_2_O, 3.00 g of (NH_4_)_2_SO_4_, 0.10 g of KCl, 0.50 g of K_2_HPO_4_, 0.50 g of MgSO_4_·7H_2_O, and 0.01 g of Ca(NO_3_)2 in 1 L of deionized water, adjusted to pH 2.5 with H_2_SO_4_. *A*. *ferrooxidans* LX5 cell suspension was prepared as reported previously [23]. The final cell density was approximately 3.0 × 10^8^ cells/mL.

### Effect of oxygen supply level (as indicated by different reaction solution loading volumes) on jarosite formation promoted by *A*. *ferrooxidans* LX5

The ferrous bio-oxidation was performed in 500 mL conical flasks, each containing 3.0 × 10^8^ cells/mL of *A*. *ferrooxidans* LX5 and different loading volumes of 9K mineral salt solutions. To achieve different DO concentrations in the reaction solutions, 100, 200, and 350 mL of 9K medium, with equivalent to 20%, 40%, and 70% of solution loading volume percentages, were added to 500 mL conical flasks, respectively. The solution pH was uniformly adjusted to pH 2.5 with H_2_SO_4_, followed by incubation at 28°C and 180 rpm in a rotary shaker. During incubation, the concentrations of DO, Fe^2+^, Fe^3+^, and the precipitates in the solutions were periodically determined. Given that the abiotic oxidation of ferrous iron is hardly initiated below pH 4.5, a control group without the inoculation of *A*. *ferrooxidans* LX5 was not designed in this study.

### Effect of limiting oxygen supply on the jarosite formation promoted by *A*. *ferrooxidans* LX5

Three groups of sealed flask experiments were performed to determine whether O_2_ was continuously required in the whole jarosite biosynthesis process. One group of flasks was not sealed throughout the jarosite formation, whereas another group of flasks was sealed at the end of the ferrous oxidation reaction (i.e., no Fe^2+^ remained in the solution). Several flasks, which were sealed by paraffin at the beginning of the ferrous oxidation reaction, were designed as the control group.

After 120 h of incubation, all precipitates that formed in the flasks were filtered through Whatman No. 4 filter paper, washed twice with distilled water, dried at 60°C to constant weight, and characterized via the methods described below.

During these trials, the water content lost in the flasks due to evaporation was replenished with distilled water by weight method. All experiments were performed in triplicate. The data were presented as the mean values of triplicate samples with standard deviations.

### Analytical methods

The DO values of the solutions were measured using a Thermo DO analyzer. The concentrations of Fe^2+^ and Fe^3+^ were determined via 1, 10-phenanthroline colorimetric method [24].

The crystal structure of the formed Fe-precipitates was examined via X-ray powder diffraction (XRD) (Rigaku Rotaflex D/max, Japan) with CuKα radiation (50 kV, 150 mA). XRD patterns were obtained by scanning speed of 6 deg/min and scanning angles in 10–80 deg. The characteristic reflection peaks (*d*-values) were matched with the peaks in the database of the Joint Committee on Powder Diffraction Standards [25].

Morphology and elemental composition were examined using a scanning electron microscope equipped with an energy dispersive spectral analyzer (EDS) [Zeiss S-EV018, Germany], operated at 20.0 kV accelerating voltage [13,23]. EDS analysis of mineral sample has typically been applied to quantitative identification of major elements with rough estimates of composition based on relative peak intensities.

## Results and Discussion

### Effects of oxygen supply level on the jarosite formation

**Fig 1:**
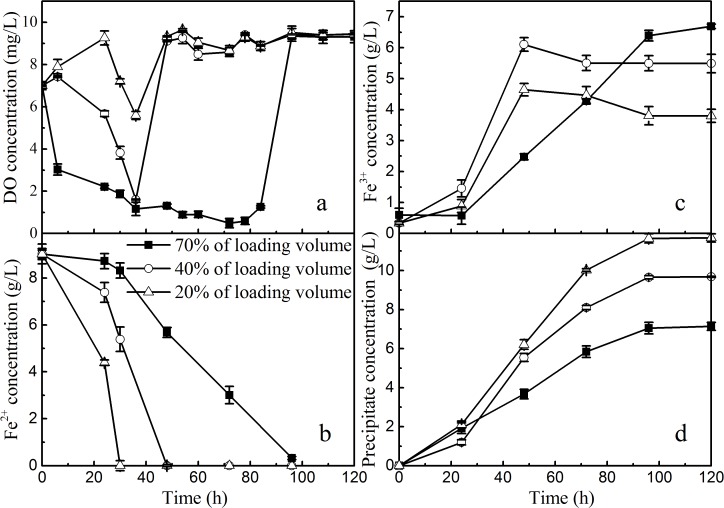
Changes in the concentrations of DO, Fe^2+^, Fe^3+^, and Fe-precipitates during the jarosite formation process with reaction solution loading volume percentages ranging from 20% to 70% (■70% of loading volume, ○40% of loading volume, Δ20% of loading volume).

Dynamic changes in the concentrations of DO, Fe^2+^, Fe^3+^, and precipitate mass with different loading volumes of the reaction solutions (20%, 40%, and 70% v/v) during the ferrous bio-oxidation and ferric precipitation periods are shown in Fig. 1. The concentrations varied drastically with increasing reaction time. For example, DO concentrations in the solution decreased rapidly in the first 40 h to 80 h because of consumption during the bacterial oxidation of ferrous iron and then increased gradually from their lowest values (5.8, 2.0, and 0.7 mg/L in 20%, 40%, and 70% of loading volume percentages, respectively) to saturate values (9.0 mg/L) because of the subsequent supplement of O_2_ from air.

A continuous decrease in Fe^2+^ concentration was observed in all three groups of reaction systems, and the decrease rate increased with decreasing solution loading volume (Fig. 1B), which was attributed to the fact that the presence of higher levels of DO in the solution was favorable for the growth of *A*. *ferrooxidans* LX5 [20,26]. Notably, for 20% solution loading volume percentage, the DO concentration was always maintained higher than 6 mg/L in the whole ferrous bio-oxidation period, and 9 g/L of Fe^2+^ was oxidized completely within 30 h, which was equivalent to a Fe^2+^ oxidation rate of 300 mg/(L∙h). This value, however, was cut in half at a solution loading volume percentage of 40%. These findings were consistent with those in previous reports [13,27,28].

Following a continuous decease in Fe^2+^ concentration, Fe^3+^ concentration in all three reaction systems gradually increased, but the increase dynamics at different loading volume conditions showed a significant difference from that of Fe^2+^ changes (Fig. 1C). When the solution loading volume percentage was decreased from 40% to 20%, an increase in the rate of Fe^3+^ production from 112.5 mg/(L∙h) to 150 mg/(L∙h) was observed, which was probably associated with the hydrolysis reaction and formation of Fe-precipitates, which are attributed to a part of the already produced Fe^3+^ [27,29,30]. In fact, when the FeSO_4_ solution became a weakly yellow turbid suspension, the precipitates formed shortly afterwards. Fig. 1D shows the precipitate contents formed at different reaction solution loading volumes. Approximately 2 g/L of Fe-precipitate was formed in the first 24 h, after which it increased rapidly with increasing reaction time, finally reaching 11 g/L, 9.5 g/L, and 7.5 g/L in 20%, 40%, and 70% loading volume percentages, respectively. This finding implied that the oxygen supply level in the reaction solution had no substantial influence on the contents of the formed Fe-precipitates.

The Pearson linear regression model was used to determine the relationships between the concentrations of DO, Fe^3+^, Fe^2+^, and Fe^2+^ oxidation rate and the formation of Fe-precipitates (Table 1). The formation of Fe-precipitates was poorly correlated with DO concentration [R^2^ = 0.158 and root mean square error (Root-MSE) = 41.42] and mainly correlated with the concentrations of produced Fe^3+^ (R^2^ = 0.952 and Root-MSE = 16.72). Similar results have been reported by Huang and Zhou [16] and Mahiroglu *et al*. [31], who found that the Fe^2+^ oxidation rate, to a great extent, had a dominant function in the formation of monomineral phase of iron hydroxysulfate.

**Table 1:** Pearson correlation between the concentrations of DO, Fe^2+^, and Fe^3+^ and the mass of produced jarosite.

Equation	R^2^	Root-MSE
Jarosite mass = 0.532 × (DO concentration)	0.158	41.42
Jarosite mass = −0.71+1.89 × (Fe^3+^ concentration)	0.952	16.27
Jarosite mass = 9.10–0.99 × (Fe^2+^ concentration)	0.881	1.63

### Effect of limiting oxygen supply on the jarosite formation

**Fig 2:**
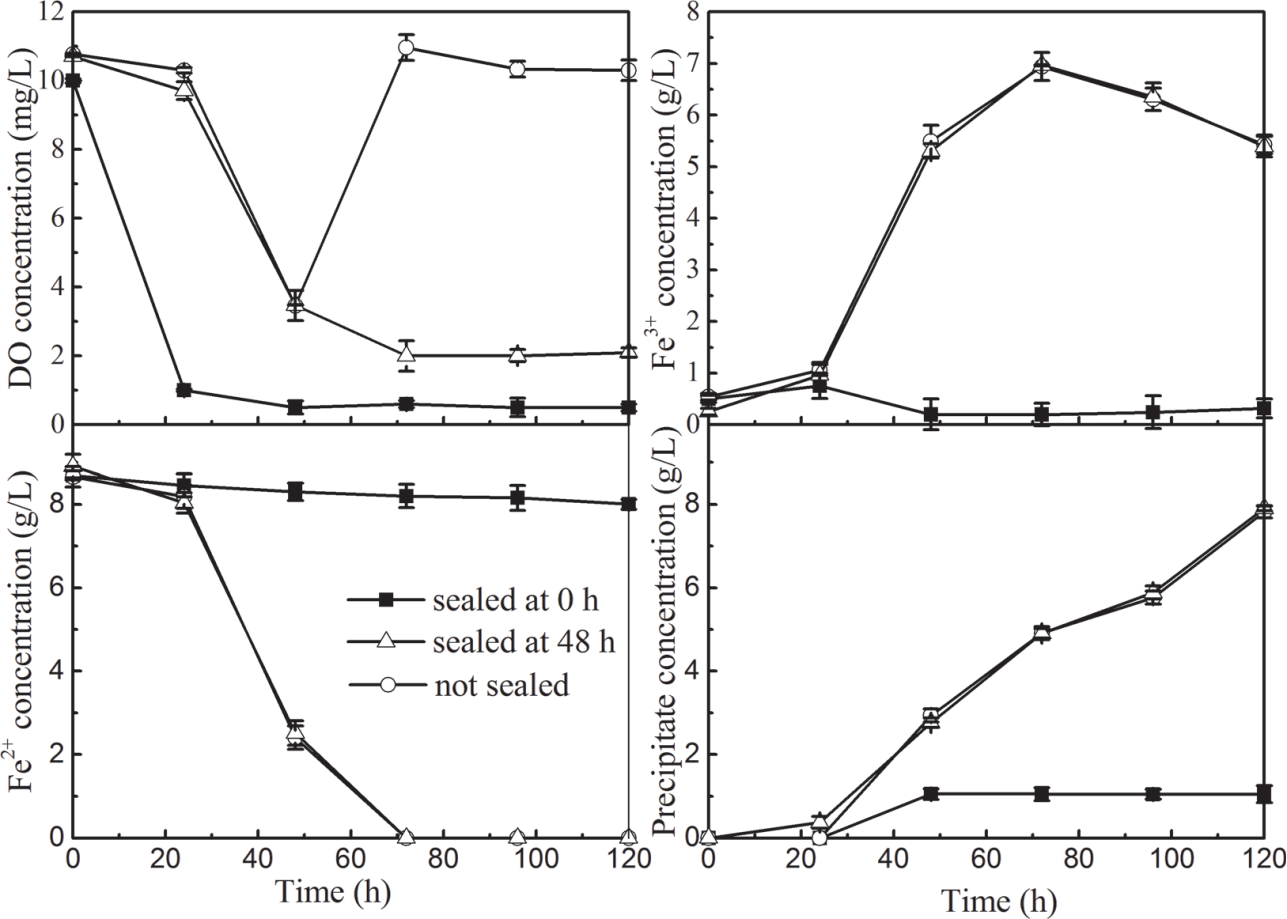
Changes in the concentrations of DO, Fe^2+^, Fe^3+^, and Fe-precipitates during the jarosite formation process with limiting oxygen supply (■sealed at 0 h, Δsealed at 48 h, ○unsealed).

To determine further the function of DO in the Fe-precipitate formation, three groups of sealed flasks experiments [i.e., flasks containing FeSO_4_ solution were not sealed by paraffin, sealed at the start of the ferrous oxidation reaction (0 h), and at the end of the ferrous oxidation reaction (48 h)] were conducted. Notably, Fig. 2 shows that DO concentration in the initially sealed reaction solution decreased sharply from 10 mg/L to 0.3 mg/L within the first 24 h and then remained at this value. Accordingly, a marked inhibition of Fe^2+^ bio-oxidation and Fe-precipitate formation was observed as indicated by low oxidation rate of Fe^2+^ (5.5%) and trace amounts of Fe-precipitates (0.65 g/L) at the termination of the reaction (120 h). This finding suggested that relying only on the DO remaining in the initial reaction solution hardly supports the O_2_ requirement for jarosite formation given that *A*. *ferrooxidans* should utilize O_2_ to oxidize ferrous iron and obtain energy for its growth [3, 32]. Meanwhile, almost the same dynamic changes in the concentrations of Fe^2+^, Fe^3+^, and Fe-precipitates were observed even though the DO concentration in unsealed flasks was always five times that in 48 h sealed flasks during the ferric precipitation period. This phenomenon suggested that the supply of oxygen had an important function in the oxidation of Fe^2+^ to Fe^3+^ by *A*. *ferrooxidans* LX5; however, its function in the formation of Fe-precipitates could be neglected possibly because the energy released from the oxidation of Fe^2+^ can be used for the subsequent hydrolysis reaction of Fe^3+^ into jarosite [33,34]. This advantage would substantially reduce the cost of commercial jarosite synthesis because the aeration operation can be eliminated in the Fe^3+^ precipitation period.

### Characteristics of Fe-precipitates

**Fig 3:**
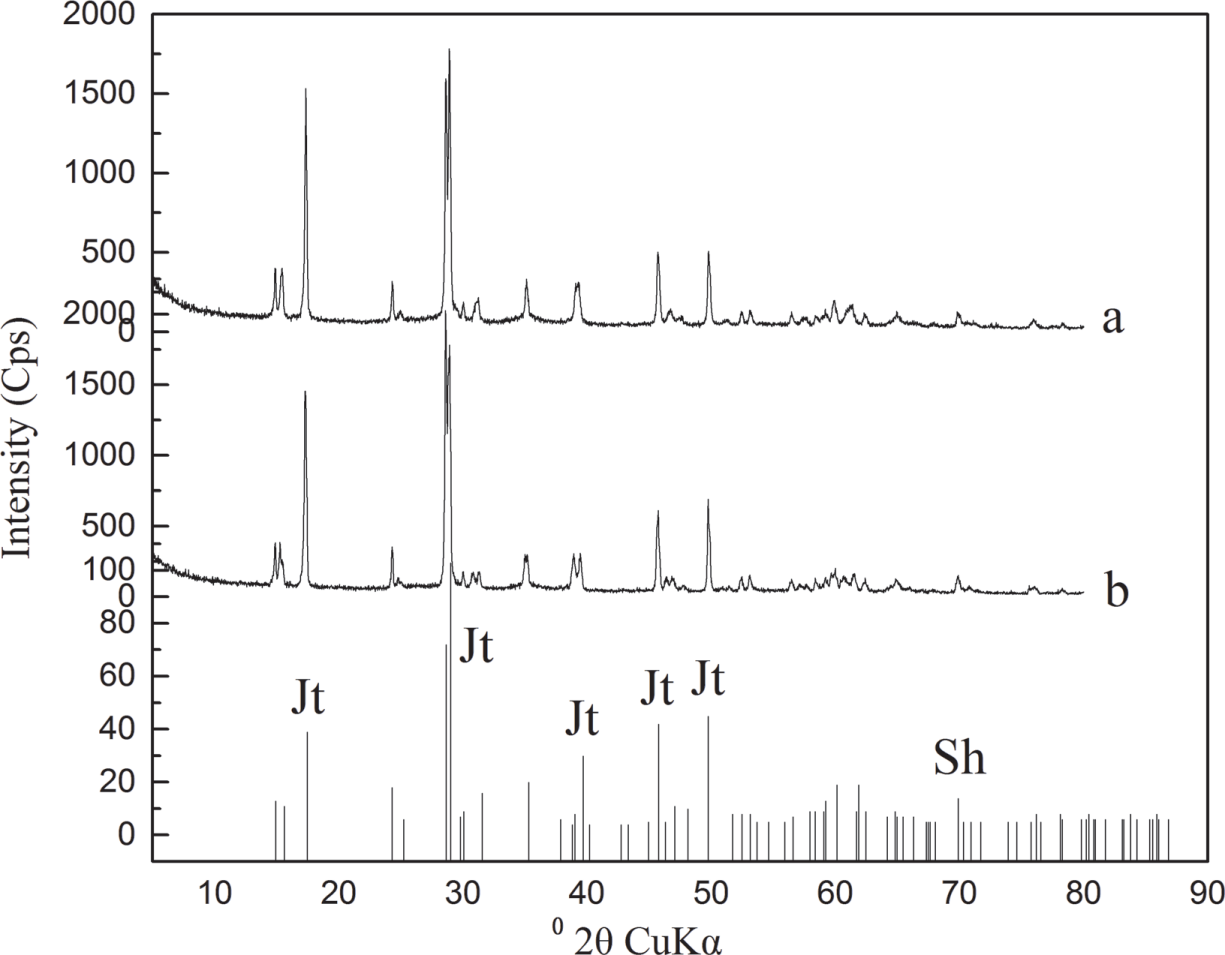
XRD patterns of Fe-precipitates formed at 48 h sealed (a) and unsealed reaction solutions (b).

The XRD results indicated that the Fe-precipitates that formed in both the unsealed and 48 h sealed groups exhibited fine crystallinity because of the presence of strong and sharp whole-angle diffraction peaks (Fig. 3). These precipitates were uniformly identified as jarosite according to the JCPDS card [26] given that no significant difference was observed between the XRD patterns of the precipitate in the present study and the standard jarosite pattern.

**Fig 4:**
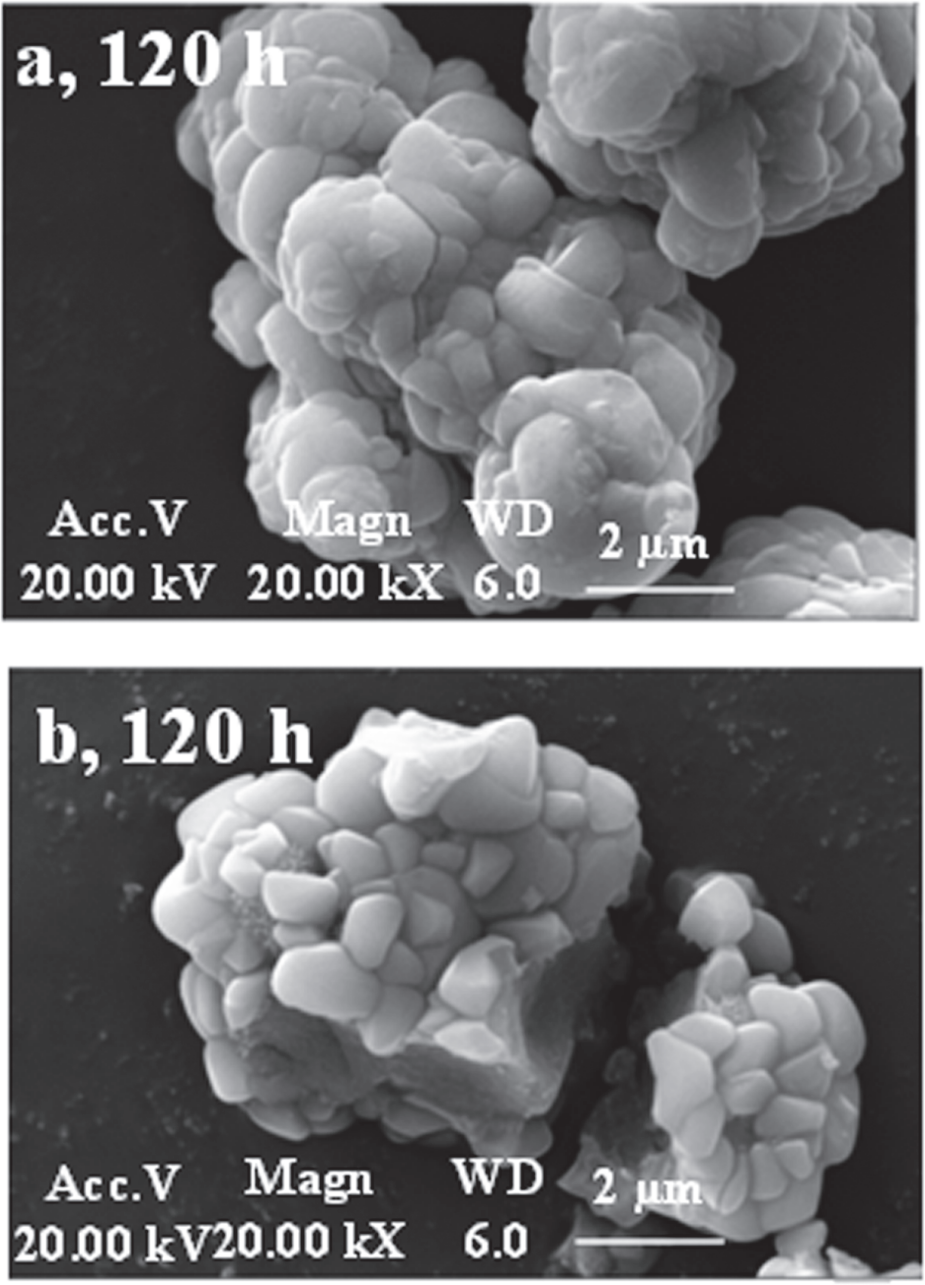
SEM images of Fe-precipitates formed at 48 h sealed (a) and unsealed reaction solutions (b).

**Fig 5:**
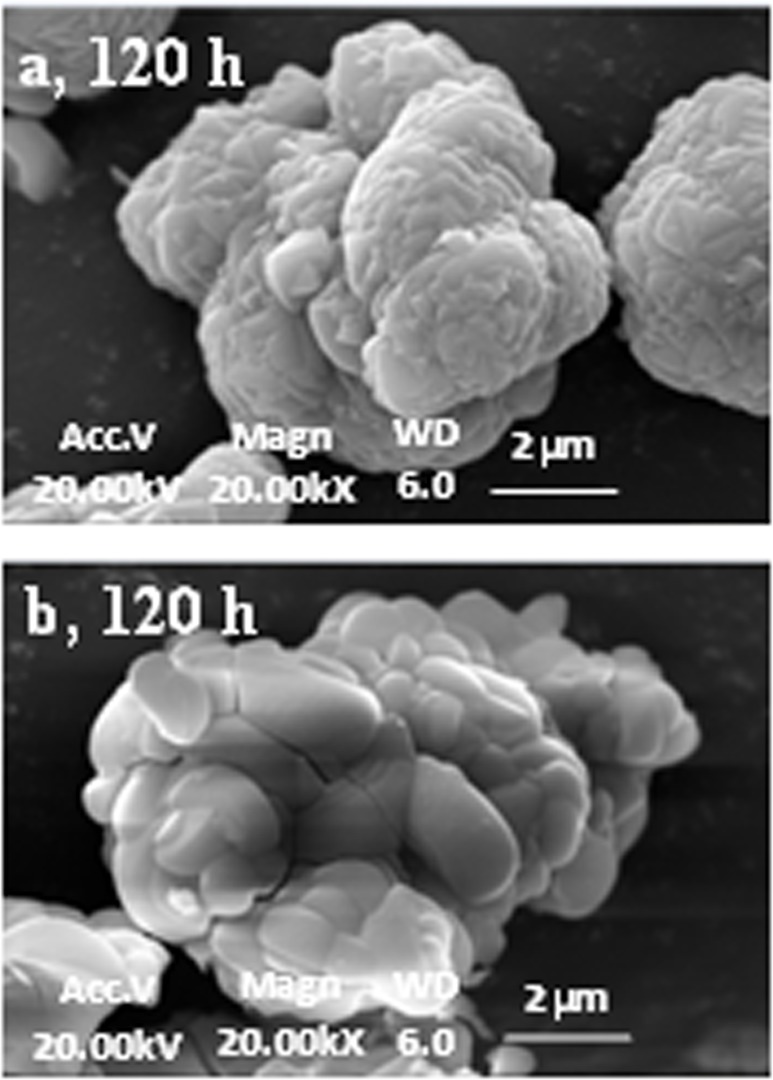
SEM images of Fe-precipitates formed in the solutions with the loading volume percentage of 20% (a) and 70% (b).

Scanning electron microscopy (SEM) images showed that irregular geometric-type jarosite particles with diameters of 1 μm to 1.5 μm and a smooth surface were dominantly formed in the unsealed and 48 h sealed groups (Fig. 4). The jarosite particles formed at different solution loading volume conditions significantly varied in both particle size and morphology (Fig. 5). Spheroid-type jarosite particles with a diameter of 6 μm and a rough surface were formed at a solution loading volume percentage of 20%. At a solution loading volume percentage of 70%, the formed jarosite particles exhibited an irregular shape, and their surfaces were relatively smoother. The difference in the morphology and size of jarosite formed at different solution loading volumes was mainly attributed to the varying shearing force in the flasks. Similar phenomena have been reported by Asokan *et al*. [6] and Elwood *et al*. [35], who found that the diameters of jarosite particles formed at different hydraulic shearing forces were in the range of 1 μm to 16.2 μm.

**Fig 6:**
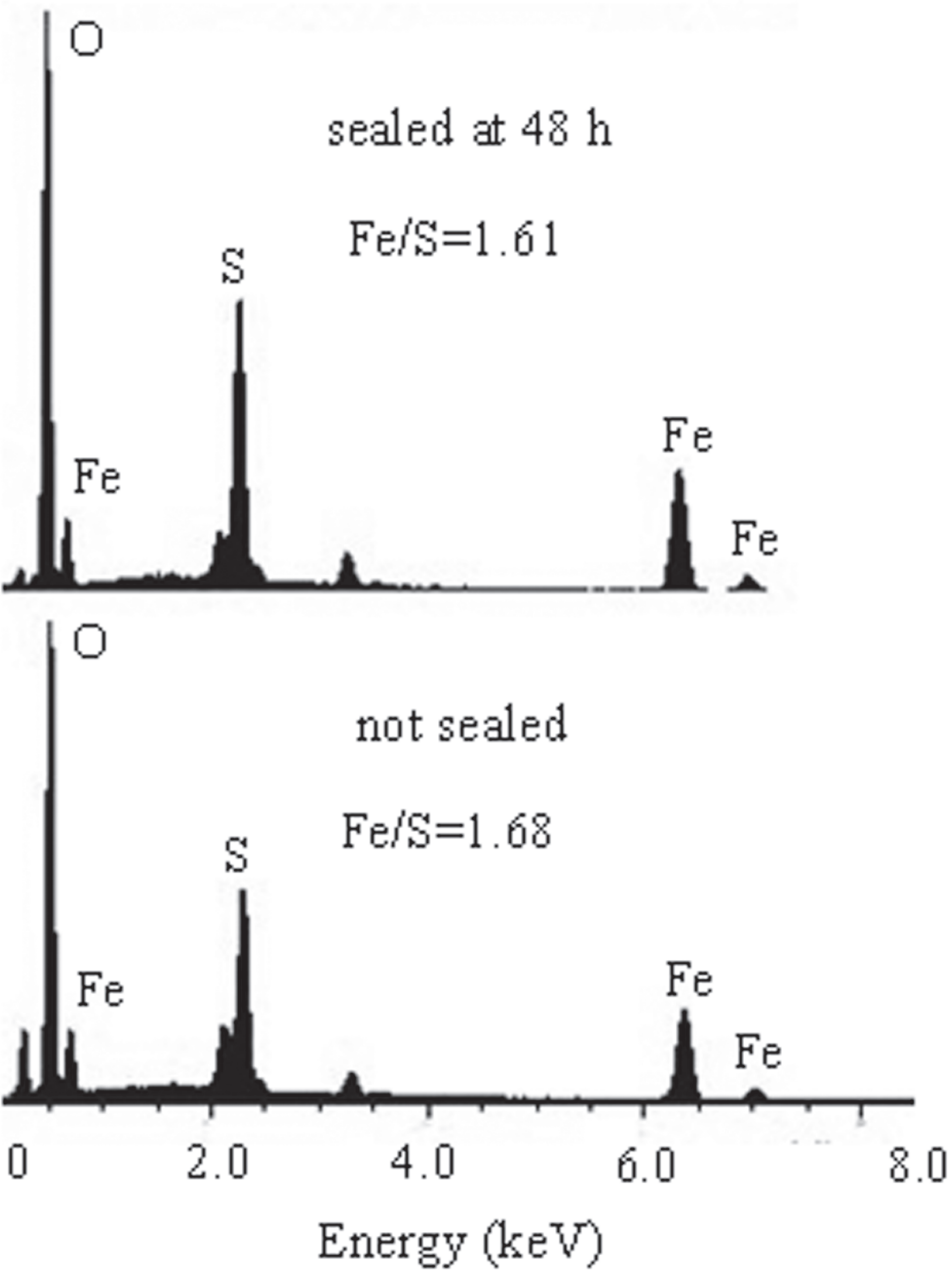
EDS analysis of Fe-precipitates formed at 48 h sealed and unsealed reaction solutions.

EDS analysis (Fig. 6) revealed that 15.3 wt% to 19.6 wt% of S and 24.6 wt% to 31.7 wt% of Fe were incorporated into the product of jarosite precipitates formed at the 48 h sealed and unsealed groups, resulting in Fe/S molar ratios of 1.61 and 1.68, respectively. The Fe/S molar ratio of jarosite obtained in the present study was consistent with the range between 1.38% and 1.8% described by Johnston *et al*. [36] and Wang *et al*. [37]. Based on these findings, we can conclude that during jarosite biosynthesis promoted by *A*. *ferrooxidans* LX5, the supply of O_2_ is only required in the oxidation of ferrous iron to ferric and did not significantly contribute to the ferric precipitation reaction.

## Conclusion

The synthesis of jarosite promoted by *A*. *ferrooxidans* LX5 can be achieved at a wide range of oxygen supply level. The rate and efficiency of jarosite biosynthesis were poorly correlated with the DO concentration in the reaction solution. The continuous supply of O_2_ was only required during the period of ferrous iron oxidation to ferric and was not required during the ferric precipitation reaction. This study provided insight into the biosynthesis mechanism of jarosite promoted by *A*. *ferrooxidans* LX5 and provides useful information on the regulation of jarosite biosynthesis.
